# The role of CaMKK2 in Golgi-associated vesicle trafficking

**DOI:** 10.1042/BST20220833

**Published:** 2023-02-23

**Authors:** Grace Kennedy, Olivia Gibson, Dáire T. O'Hare, Ian G. Mills, Emma Evergren

**Affiliations:** 1 Patrick G Johnston Centre for Cancer Research, Queen's University Belfast, 97 Lisburn Road, BT9 7AE Belfast, U.K.; 2 Nuffield Department of Surgical Sciences, University of Oxford, John Radcliffe Hospital, Headley Way, OX3 9DU Oxford, U.K.

**Keywords:** calmodulin, Golgi apparatus, protein–serine–threonine kinases, trafficking

## Abstract

Calcium/calmodulin-dependent protein kinase kinase 2 (CaMKK2) is a serine/threonine-protein kinase, that is involved in maintaining various physiological and cellular processes within the cell that regulate energy homeostasis and cell growth. CaMKK2 regulates glucose metabolism by the activation of downstream kinases, AMP-activated protein kinase (AMPK) and other calcium/calmodulin-dependent protein kinases. Consequently, its deregulation has a role in multiple human metabolic diseases including obesity and cancer. Despite the importance of CaMKK2, its signalling pathways and pathological mechanisms are not completely understood. Recent work has been aimed at broadening our understanding of the biological functions of CaMKK2. These studies have uncovered new interaction partners that have led to the description of new functions that include lipogenesis and Golgi vesicle trafficking. Here, we review recent insights into the role of CaMKK2 in membrane trafficking mechanisms and discuss the functional implications in a cellular context and for disease.

## Introduction

Calcium (Ca^2+^) functions as one of the most critical second messengers that regulate a host of downstream signalling events. The Ca^2+^ sensor calmodulin binds and regulates the activity of target proteins, which include calcium/calmodulin-dependent kinases (CaMK). CaMK kinase (CaMKK) 2 regulates CaMKI and IV by phosphorylating them. In addition, it phosphorylates the α-subunit of AMP-activated protein kinase (AMPK). AMPK is regarded as the master metabolic protein kinase and consequently CAMKK2 has emerged as the primary CaMK participating in the regulation of metabolic homeostasis. CaMKK2 is highly expressed in the hypothalamus and plays a fundamental role in whole-body energy homeostasis and glucose metabolism and is associated with many metabolic diseases. It is overexpressed in and promotes prostate, breast, liver, ovarian and gastric cancer [1–5]. In this review, we discuss research on CaMKK2 that contextualises its functions that are associated with intracellular membrane trafficking.

### Structural and functional insights to CaMKK2

**Figure 1.**
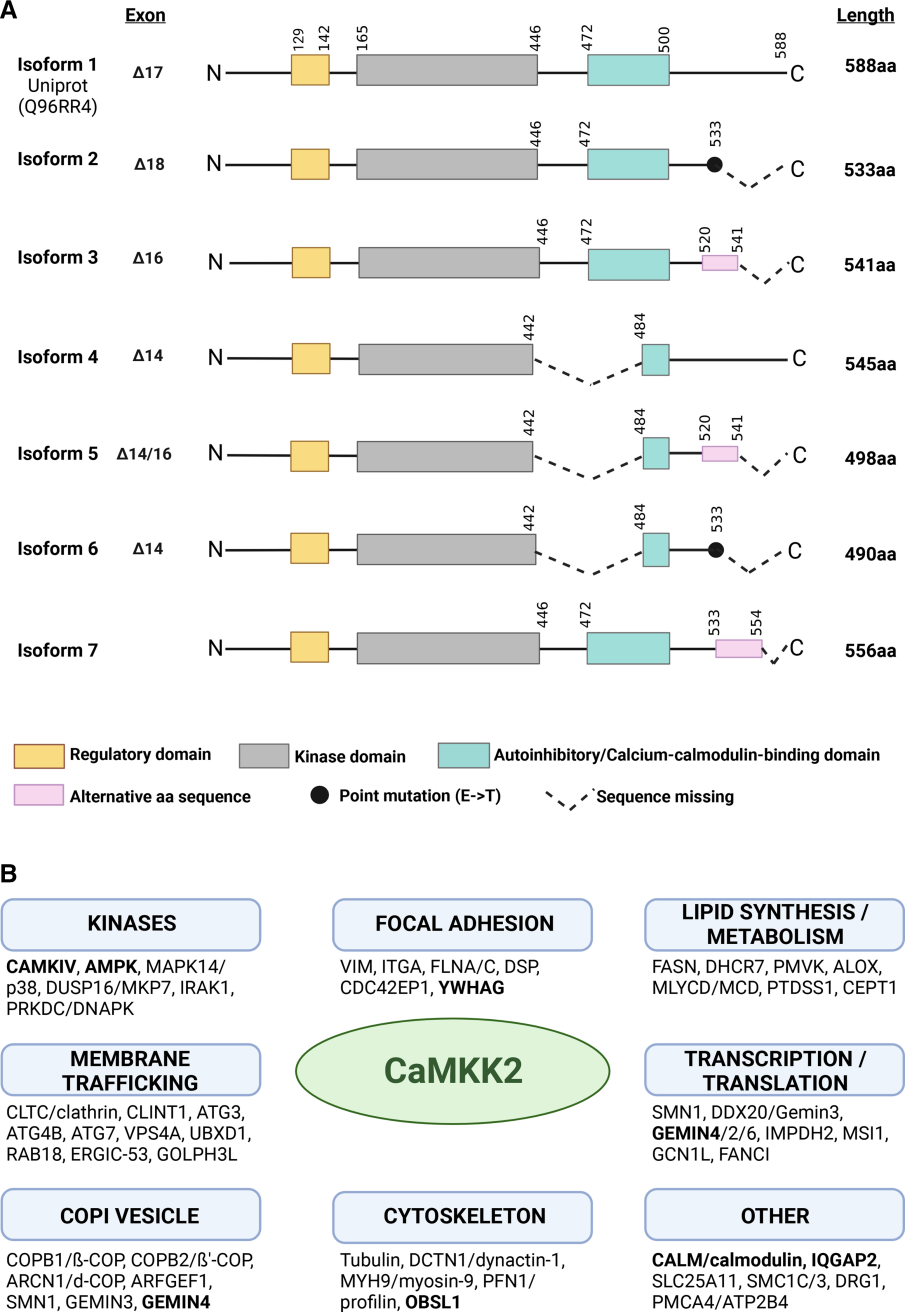
Summary of CaMKK2 domain structure and interactors identified by affinity capture mass spectrometry. (**A**) CaMKK2 contains three domains: the regulatory domain, the central serine/threonine kinase domain and the N-terminal autoinhibitory and calcium/calmodulin-binding domain (AID/CBD). There are seven distinct isoforms of CaMKK2 that differ in the C-terminal region due to the splicing of exons. Isoforms 4, 5 and 6 lack exon 14, which encodes the sequence within the AID/CBD that is phosphorylated and facilitates kinase activation. This loss removes the catalytic activity of isoforms 4, 5 and 6 and these, therefore, are found to be inactive. Isoforms 2 and 6 have truncated C-termini that may affect the inherent kinase activity. Furthermore, exon 17 is absent from isoform 1 and exon 18 is absent from isoform 2. Isoforms 3 and 5 have a deletion of exon 16 resulting in a change in the open reading frame, producing an alternative sequence (KPTRECESLSELKEARQRRQPP) from the original isoform 1 sequence (QGSEDNLQGTDPPPVGEEEVLL) and an early termination of the transcript [13]. Isoform 3 is the predominant form of CaMKK2 expressed in prostate cancer [95]. (**B**) A summary of interaction partners of CaMKK2 that have been identified by affinity capture mass spectrometry is displayed in functional categories which illustrate the range of interactions associated with CaMKK2. Protein interactions that have been validated by independent biochemical experiments are displayed in bold. Figure created with BioRender.com.

CaMKK2 (CAMKKβ) is a calcium/calmodulin-dependent serine/threonine-protein kinase [6]. The sequence homology of CaMKK2 with family members CaMKK1 (CAMKKa), CaMKI and CaMKIV are high. For example, CaMKK1 and CaMKK2 show a 70% amino acid identity [6]. CaMKK2 is composed of an N-terminal regulatory domain containing three phosphorylation sites (serines) for GSK3, CDK5 and PKA (Figure 1A). A central catalytic domain is immediately followed by a domain containing the regulatory and calmodulin-binding sequences [7]. CaMKK2 has a significant kinase activity at steady state, but its activity is significantly increased by an elevation in intracellular Ca^2+^ and binding of calcium–calmodulin (CaM) [6–8]. The interaction of CaMKK2 with CaM releases the binding of the autoinhibitory region and leads to the activation of the enzymatic activity [9,10]. Its autonomous activity is dependent on the autophosphorylation of Thr482 in the C-terminal regulatory domain, while the calmodulin-dependent activity is dependent on the phosphorylation of serine residues in the N-terminal regulatory domain [11]. The catalytic domain is composed of two lobes that are connected by a flexible linker: the ATP-binding pocket and the substrate-binding domain [12]. CaMKK2 has seven isoforms that differ in their domain composition and ability to autophosphorylate and phosphorylate downstream targets (Figure 1A). It is mainly the longest isoform that is well characterised (588 aa, 64.7 kDa). Based on the sequences (Figure 1A), there may be differences among the isoforms in relation to kinase activity, autoinhibition and autophosphorylation [1,13].

The active CaMKK2 directly phosphorylates downstream kinases CaMKI, CAMKIV and AMPK, which regulates cellular metabolism. AMPK is central in the regulation of energy homeostasis because it acts as an energy sensor. The conversion of ATP to AMP/ADP due to energy stress activates the kinase activity of AMPK, phosphorylating downstream targets that control cell proliferation pathways, and transcription and translation factors. In turn, processes like autophagy and catabolism of fatty acids and glucose are increased, while the synthesis of macromolecules like proteins and cholesterol is decreased. CaMKIV activation results in the activation of transcriptional programmes that regulate the expression of proteins that mediate cell growth and metabolism [2,14].

Further functional insights for CaMKK2 arise from studies investigating the localisation of the enzyme. Its localisation is predominantly cytosolic as determined by immunocytochemistry and biochemical fractionation experiments [15–17]. However, it has also been shown to localise to the nucleus and facilitate transcriptional regulation in prostate and ovarian epithelial cells [8,18]. In ovarian cancer cells, this translocation is induced by epidermal growth factor (EGF) stimulation [8]. In addition to its cytosolic localisation, an experiment using subcellular fractionation of endothelial cells showed a prominent association of CaMKK2 with the endoplasmic reticulum (ER) and mitochondria [15]. While more research is required to fully understand the recruitment and function of CaMKK2 at the ER and mitochondria it is interesting in the context of recent studies showing that AMPK, considered to be a mainly cytosolic protein, under some circumstances is activated on the surface of the lysosome by LKB1 and at the ER and Golgi by CaMKK2 [19–22]. Thus, illustrating the necessity to define protein–protein interactions and their spatial location within cells in order to develop a complete picture of the biological functions of CAMKK2 and other kinases in relation to signalling and metabolism.

### The CaMKK2 interactome and substrates

The interactome of CaMKK2 has been determined experimentally from co-immunoprecipitation of EGFP-tagged CaMKK2 in HEK293 cells and from immunoprecipitation of endogenous CaMKK2 from cell lines [23–26]. Among the cellular ontologies that are enriched in the interactome of CaMKK2 we find focal adhesions, lipid synthesising and metabolising enzymes, translational regulators, membrane trafficking and vesicle-associated proteins (Figure 1B). This indicates that we have a limited understanding of the function of CaMKK2, and there are additional pathways beyond cellular signalling that are regulated by CaMKK2. One example of a CaMKK2-binding protein that was identified by mass spectrometry and has expanded our understanding of the functional repertoire of CaMKK2 is fatty acid synthase (FASN) [24]. Functional studies show that the down-regulation of CaMKK2 by microRNA and in knockout mice generates a significant increase in lipid accumulation along with increased levels of FASN and SREBP1c, indicating either a direct or indirect relationship between FASN and CaMKK2 signalling [27,28]. Mapping of the kinome of CaMKK2 using quantitative phospho-proteomics of cells treated with the inhibitor STO-609 also enhance our understanding of the function of the protein. Recent studies in gastric cancer cell lines identified several potential novel substrates of CaMKK2 [29,30]. Novel CaMKK2 substrates include kinases in the ephrin receptor signalling pathway, EGF signalling and the MAPK pathway. Furthermore, proteins that are hypo-phosphorylated in cells treated with STO-609 associate with focal adhesions, plasma membrane, cytosol, vesicle membrane and the Golgi apparatus to mention a few [29]. The main limitation of these studies is the poor specificity of the inhibitor STO-609, but nevertheless adds significant information to our understanding of the complexity of CaMKK2 functions [10]. Together, the interactome and kinome of CaMKK2 implicate that this kinase has functions associated with membranes and membrane trafficking.

### Regulation of intracellular membrane trafficking by calcium/calmodulin

Cells rapidly respond to their physical environment, energy demands and signalling molecules. A key part of this response is the use of vesicle trafficking between organelles and in the secretory pathway. These trafficking pathways are important for the delivery of newly synthesised proteins and lipids to the correct target location, and in addition, orchestrate the homeostasis of these organelles. Here, we will discuss their regulation by CaM. The main calcium stores in the mammalian cell are the ER, Golgi and the lysosome [31]. Calcium plays a well-established role in vesicle trafficking and CaM is emerging as an additional regulatory complex [32,33]. The direct involvement of CaM in endocytosis has recently been demonstrated through experiments in the calyx of Held nerve terminals in CaM knockout mice [34]. The effectors of CaM that regulate endocytosis include calcineurin, a protein phosphatase, CaMKK2 and protein kinase C (PKC) [34–36]. Vesicle trafficking between the ER and Golgi is regulated by calcium through mechanisms that include Ca^2+^ channels in the ER [31]. Vesicle formation is tightly regulated by the recruitment of membrane-binding and structural proteins that together form a coat of a defined size that envelope the membrane and create a bud that can be pinched off from the organelle. There are classes of coat proteins that facilitate the formation of vesicles from different organelles. For example, COPI and COPII, on the Golgi and ER mediate the trafficking of cargo in the retrograde and anterograde direction. Disruption of Ca^2+^ signalling abrogates coat recruitment and vesicle formation for both anterograde and retrograde vesicle trafficking between these two organelles [37–40]. In this context, it is important to mention that the Golgi is a Ca^2+^ storage organelle with a concentration gradient that ranges from 300 µM in the cis-Golgi to 100 µM in the trans-Golgi [31]. A transient localised increase in Ca^2+^ concentration can be sensed by CaM (*K*_d_ = 0.5–5 µM), which results in a conformational change and interaction with target proteins [41]. Experiments using cell-free assays with purified organelles demonstrate that the calcium chelator BAPTA or CaM inhibitors strongly inhibit Golgi vesicle transport [42]. The addition of BAPTA to a cell-free system showed a distinct loss of coated vesicles subsequent to coat assembly, demonstrating that calcium or a calcium-dependent sensor stabilises the COPI coat at the Golgi membrane [39]. Together with the fact that Golgi calcium stores are significant and contribute to subcellular signalling events highlight the significance of calcium and CaM in regulating Golgi vesicle trafficking [39,43,44]. The calcium sensors and CaM-dependent effectors remain not well understood and more research is, therefore, required in this area.

### Golgi membrane trafficking

**Figure 2.**
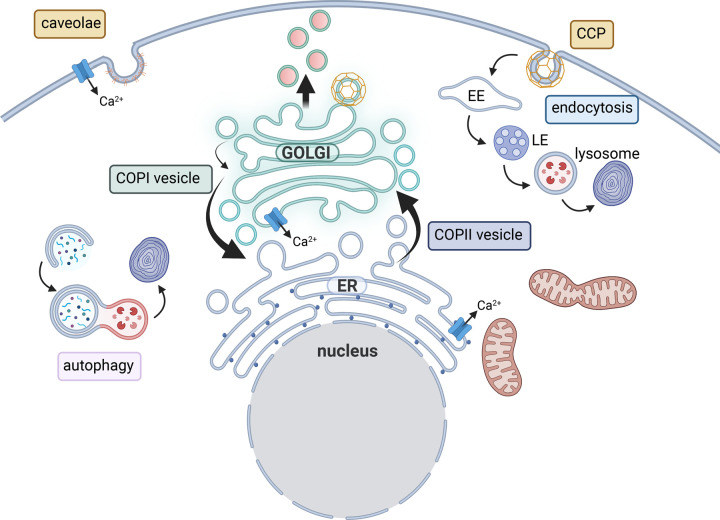
Multiple intracellular membrane trafficking pathways are relevant for CaMKK2 biology. The Golgi is central to membrane trafficking and controls protein modifications and secretion. The Golgi is an important reservoir for Ca^2+^ together with the endoplasmic reticulum (ER) and regulates ion homeostasis. The Golgi is tightly linked with other organelles; the ER, lysosome and endosomes. Golgi vesicle trafficking is mediated by three types of coated vesicles. Anterograde trafficking from the ER to the Golgi in the secretory pathway is mediated by COPII-coated vesicles. Retrograde transport of vesicles Golgi-to-ER is mediated by COPI-coated vesicles. COPI vesicles also mediate intra-Golgi transport between cisternae. Clathrin-coated vesicles bud from the trans-Golgi where cargo molecules are concentrated in these vesicles. At the plasma membrane cargo molecules and receptors are internalised by coated vesicles that include caveolae and clathrin-coated pits (CCPs). Figure created with BioRender.com.

The Golgi supports cellular functions such as intracellular vesicle trafficking, protein secretion, regulation of ion homeostasis and glycosylation [45]. It has an essential role as an intracellular signalling platform and a stress sensor that facilitates intracellular communication that impact on cell metabolism, death, migration and proliferation [46–50]. To sustain the function and organisation of Golgi cisternae and its resident proteins it is important that the flux of anterograde and retrograde vesicle trafficking is in balance [51,52]. Three types of coated vesicles have been described in association with the Golgi; COPI, COPII and clathrin [53–55] (Figure 2). Clathrin-coated vesicles bud from the trans-Golgi and mainly transport enzymes to the lysosome [56]. COPII-coated vesicle mainly transport cargo from the ER to the Golgi, while COPI-coated vesicles facilitate retrograde traffic from the Golgi to the ER and within the Golgi (Figure 2). Vesicle formation is modulated by the conserved oligomeric Golgi (COG) complex and small nucleotide-binding proteins of the Rab and Arf families that facilitate the recruitment of coatomer to the membrane [57]. The interactions of the coatomers with these modulators are important for protein stabilisation and vesicle trafficking [58]. Together, this illustrates the close relationship between modulating proteins and the impact on protein stability and functional Golgi vesicle trafficking. The Golgi is organised in stacks of cisternae with a *cis*- and a *trans*-face. These mini-stacks are linked together in a ribbon structure and can undergo dynamic changes resulting in compaction, dispersal, disassembly of the ribbon or dispersal of both the ribbon and mini-stacks [59]. Golgi morphology may change due to the dispersal of one compartment, for example, the trans-Golgi cisternae. Golgi morphology is regulated by kinases and phosphatases, making it suited to respond to and co-ordinate cell signalling and processes during cancer and stress conditions [60]. Pathological conditions like these may result in the loss of the Golgi ribbon, which leads to a dispersed appearance [59]. For example, loss of α-COP, a COPI coatomer or phosphorylation of Rab7 results in a dispersed Golgi morphology and increased area of the trans-Golgi [61,62]. An altered Golgi morphology is often observed when proteins linking the cisternae or the ribbon are depleted or the interaction with the cytoskeleton is lost, but is also regulated by Rab GTPases [60,63–66].

Given the central role of the Golgi and Golgi-ER trafficking in supporting organelle biogenesis and enzyme activity, it will also play an important role in responding and adapting to metabolic and proteotoxic stress. The ER responds through the unfolded protein response which can enhance protein folding capacity but also autophagic flux. This also leads to significant changes in the trafficking of vesicles between the Golgi and the ER and in protein secretion. To support these changes Golgi proteins are up-regulated and some of these are structural or mediators of vesicle trafficking [67–70]. Recent studies have linked the Golgi apparatus as an organelle able to control mTOR signalling directly, in addition to the tight regulation of mTOR signalling and cell proliferation by the lysosome [71–73]. One of the studies discovered part of ER-to-Golgi trafficking machinery, Rab1A, to be an independent controller of mTORC1 promoting cell proliferation [74]. We recently undertook a study to learn more about the role of CaMKK2 in prostate cancer which commenced with a characterisation of CaMKK2 protein interactions and led us to investigate effects on Golgi trafficking and lysosome function in cancer cell proliferation.

### CaMKK2 as a regulator of membrane trafficking events

**Figure 3.**
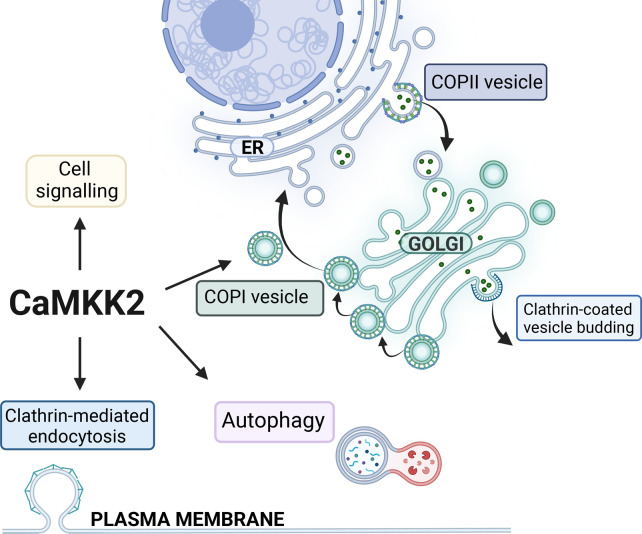
CaMKK2 in intracellular membrane trafficking. Activation of CaMKK2 via its interaction with Ca^2+^/calmodulin results in the activation of downstream signalling pathways through kinases AMPK, CaMKI, CaMKIV and protein kinase B (PKB). This drives functions related to metabolic regulation and transcriptional regulation. In addition to these activities, CaMKK2 regulates multiple membrane trafficking pathways, which include autophagy, clathrin-mediated endocytosis and Golgi vesicle trafficking. Regulation of these membrane trafficking pathways is important for cellular control of metabolism and cell survival. Figure created with BioRender.com.

Recently, a few studies have shown an association of CaMKK2 with membrane trafficking events; endocytosis and COPI trafficking (Figure 3) [25,75]. A genome-wide screen of kinases that regulate transferrin internalisation identified CaMKK2 as a regulator of endocytosis [36]. Further studies show that a loss of CaMKK2 expression leads to impaired transferrin internalisation in HepG2, LNCaP and HeLa cell lines, which results in reduced intracellular iron content and impaired glucose metabolism and glycolysis [15,25,36,76]. Whether CaMKK2 directly regulates adaptor or accessory proteins in clathrin-mediated endocytosis, the endocytic pathway for the Transferrin receptor, is not known. In addition, CaMKK2 associates with the Cav1.2 calcium channel at caveolae on the plasma membrane where it phosphorylates CaMKI upstream of the transcription factor CREB, which results in the regulation of chemotaxis in vascular myocytes [75]. This illustrates the significance of membrane-associated localisation and temporal Ca^2+^ activation of CaMKK2. Co-immunoprecipiation studies have shown that CaMKK2 associates with the δ-COP coat protein, a part of the COPI vesicle coat that mediates Golgi-ER trafficking [24,25]. This observation was further validated by demonstrating that the CaMKK2–Gemin4 complex efficiently enriched COPI coat proteins from a cell lysate [25]. Gemin4 is a part of multiple protein complexes that are involved in COPI/coatomer formation, autophagy, assembly of small nuclear ribonucleoproteins and trafficking of ribonucleoproteins. The COPI coat is composed from seven proteins that assemble into triad units that are recruited to the membrane together and interact with up to four triads to build the lattice of the coat [77,78]. The flexible linker regions of δ-COP and α-COP are important for cross-linking the coatomer triads to stabilise the lattice. The binding of ER retrieval signals by α-, β- and δ-COP further facilitates molecular rearrangements that stabilises the COPI coat [53,77,79–82]. Knockdown of CaMKK2 in the prostate cancer cell line LNCaP results in a significant reduction in δ-COP protein levels, along with a reduction in α-COP [25]. Furthermore, inhibition of the kinase activity of CaMKK2 reduces the levels of δ-COP to similar levels [25]. This could be due to transcriptional changes, instability of the protein complex due to loss of a direct protein interaction or regulation by phosphorylation, consequently more experiments are required to elucidate this mechanism. The gene encoding δ-COP is transcriptionally regulated by the androgen receptor, which makes it more likely to be due to protein stability. The reduction in protein expression of coatomer, in particular δ-COP that cross-links the coat proteins, would be expected to negatively impact on the stability of COPI coats and the productive vesicle trafficking events in CaMKK2 knockdown cells, resulting in impaired membrane trafficking and organelle homeostasis. The study observed an enlarged Golgi, ER stress, impaired lysosomal acidification and reduced clathrin-mediated transferrin endocytosis [25]. These membrane trafficking pathways are interconnected due to the topological relationship between these organelles. A loss of transferrin uptake can be due either to a loss of assembly of the clathrin coat at the plasma membrane or a loss of intracellular membrane trafficking. For example, a reduction in β1-COP expression results in a reduction in transferrin uptake [83]. Furthermore, loss of membrane homeostasis in the ER-Golgi also impacts on the lysosomal maturation and biogenesis [84–87]. In cells where CaMKK2 expression is knocked down the acidification of lysosomes is significantly reduced, the sensitivity to BafilomycinA treatment impaired and the processing of the lysosomal protein CathepsinD reduced [25]. This indicates that membrane trafficking through the Golgi is slowed, which creates an unbalance in the membrane trafficking of the endomembrane compartments. The COPI coatomer complex, and in particular δ-COP, is not only required for retrograde vesicle trafficking but also essential for productive autophagy [88–90]. Autophagy is an important mechanism in cancer cells that are under metabolic stress, which produces energy and amino acids necessary for cell survival. Experiments in multiple cancer cells show that COPI vesicle trafficking is not only facilitates Golgi to ER trafficking but also mediates traffic within the ER and to lysosomes and regulates autophagy [73,88,91–94]. The loss of COPI coat components results in impaired cell survival and an increased ER stress, which may be due to the disrupted trafficking leading to an accumulation of proteins in the ER and an activation of the unfolded protein response [88]. This phenotype is also observed upon loss of CaMKK2 expression in a prostate cancer cell line [25], suggesting that the observed accompanying loss of COPI coat proteins may cause of the impaired autophagy and ER stress observed thus further illustrating the significant role of CaMKK2 in Golgi vesicle trafficking and cell survival.

### Implications for our understanding of how CaMKK2 contributes to cancer progression

CaMKK2 is known to be significantly overexpressed in many cancers, including prostate, glioblastoma, liver and ovarian cancer [2,8,18,95,96]. Given the significant literature on the role of AMPK and CAMKIV activities on metabolic regulation, and their established status as CaMKK2 substrates, many of the biological effects of CaMKK2 on cancer have been attributed to this relationship. Our recent study suggests that there are fundamental roles for CaMKK2 as a regulator of membrane trafficking that can have equally profound effects on the metabolic and hence the proliferative capacity of cancer cells [25,97,98]. We know from the work of Michael White and others that COPI coatomer expression and retrograde Golgi trafficking sustains lysosomal activity and metabolic function in LKB1-mutant lung cancers [88,99]. We also know that increased COPI coatomer subunit expression is a cancer marker [88,100]. Our work suggests that CaMKK2 activity supports the same tumorigenic biology. Translationally future studies will need to address whether CaMKK2 activity or expression is a hallmark of the same coatomer-dependent sub-types of cancer and biologically future studies will need to determine what the CaMKK2 substrates are that regulate COPI coatomer subunit expression or stability. Finally given the inability to rescue defects induced by targeting CaMKK2 with either CAMKIV overexpression in liver cancer or with AMPK activators in prostate cancer [28], it will be important to determine whether COPI coatomer subunit overexpression can rescue defects in these or other disease settings. Cell biology and close attention to the spatial context in which enzymes function can provide new insights into the functions of long-studied kinases in health and disease.

## Conclusion

Our understanding of the functions of CaMKK2 in health and disease has progressed significantly recently through studies of the interactome, kinome, development of new inhibitors and the study of phenotypes and mechanisms in knockdown cells and knockout animals [1,25,28,75,88,101–105]. We have started to uncover new ways in which CaMKK2 regulates energy homeostasis, cell proliferation and migration. In doing so we find that CaMKK2 associated with membranes; plasma membrane, autophagic vacuoles, Golgi apparatus and peroxisomes; regulates novel mechanisms that are relevant for our understanding of CaMKK2 in disease biology and helps us to interpret the effects of novel CaMKK2 inhibitors. We are just starting to appreciate the various functions that CaMKK2 has and how some of these may be cell-type specific and contribute to disease biology [25,75,103].

## Perspectives

- *Importance of the field*: The Golgi apparatus is central to fundamental cellular processes such as protein trafficking, cell proliferation and ion homeostasis. It is, therefore, critical to understand the modulators that regulate the vesicle trafficking that maintains its morphology and function. CaMKK2 is a novel modulator of Golgi vesicle trafficking that is overexpressed in several diseases.
- *Current thinking*: Golgi-mediated cell communication is important for the regulation of cell metabolism, migration and proliferation. Modulators of Golgi vesicle trafficking are important for stabilising the coat components and modulating vesicle trafficking and cellular functions linked to cancer development.
- *Future direction*: Translational research studies will be important to identify sub-types of cancers that are dependent on CaMKK2 and COPI trafficking. The molecular mechanisms that contribute to the stabilisation of coatomer should also be actively investigated as these appear to promote pro-proliferative mechanisms in cancer.

## Open Access

Open access for this article was enabled by the participation of Queen's University Belfast in an all-inclusive *Read & Publish* agreement with Portland Press and the Biochemical Society under a transformative agreement with JISC.

## Abbreviations

AMPK: AMP-activated protein kinase
Ca^2+^: calcium
CaM: calcium and calmodulin
CaMKK2: calcium/calmodulin-dependent protein kinase kinase 2
COG: conserved oligomeric Golgi
EGF: epidermal growth factor
ER: endoplasmic reticulum
FASN: fatty acid synthase
PKC: protein kinase C
